# Serum Levels of Irisin and Omentin-1 in Breast Neoplasms and Their Association with Tumor Histology

**DOI:** 10.1155/2021/6656671

**Published:** 2021-02-22

**Authors:** Grigorios Panagiotou, Sofia Triantafyllidou, Basil C. Tarlatzis, Eleni Papakonstantinou

**Affiliations:** ^1^First Department of Pharmacology, School of Medicine, Aristotle University of Thessaloniki, Thessaloniki, Greece; ^2^Breast Cancer Clinic, Theagenio Cancer Hospital, Thessaloniki, Greece; ^3^Unit of Human Reproduction, 1st Department of Obstetrics & Gynecology, Papageorgiou General Hospital, School of Medicine, Aristotle University of Thessaloniki, Thessaloniki, Greece

## Abstract

Breast cancer is associated with obesity, possibly due to direct effects of adipokines and myokines, such as omentin-1 and irisin. In this study, we aimed to evaluate omentin-1 and irisin levels in women with benign and/or malignant breast neoplasms vs. healthy controls. Disease-free individuals (*N* = 56) and patients with histologically proven benign (*N* = 61) or malignant tumor (*N* = 96; subdivided into recently diagnosed/treatment-naïve (*N* = 72) and chemotherapy-treated (*N* = 24) subgroups) were enrolled in this study. Demographic, biochemical, and tumor histological characteristics were recorded. Body composition parameters were assessed using bioelectrical impedance. Serum irisin and omentin-1 levels were quantified with ELISA kits. In adjusted models, irisin levels were higher in both benign and malignant cases compared to controls but were comparable between neoplasms. Further adjustment for omentin-1 levels showed that age (odds ratio (OR) = 1.05, 95% confidence interval (95% CI) = (1.02, 1.08), *p* < 0.01) and irisin levels (OR = 5.30, 95% CI = (1.24, 22.38), *p*=0.03) were independent predictors of the presence of malignancy. These molecules were associated with each other and with other anthropometric and demographic parameters. Irisin was associated with tumor histological characteristics including Ki67% levels, Elston-Ellis grading system, and estrogen receptors status. Omentin-1 was also associated with the Elston-Ellis grading system. In conclusion, serum irisin is increased in patients with both benign and malignant diseases of the breast. When combined with omentin-1, irisin concentration was associated with the presence of breast malignancy. This molecule's role as a potential diagnostic and/or prognostic agent in breast malignancies warrants further investigation in larger prospective studies.

## 1. Introduction

The modern epidemic of obesity has been associated with various comorbidities, including diabetes mellitus, cardiovascular diseases, and cancer [1]. During recent years, an increasing number of malignancies of different origins, including colon, endometrial, and postmenopausal breast cancer were shown to be associated with body fat accumulation and sedentary lifestyle [2]. It is reported that moderate exercise may decrease breast cancer risk up to 20–30% [3].

One of the proposed mechanisms to link obesity, lack of exercise, and tumor development is the direct effects of adipose tissue secreted molecules, named adipokines, which are altered in the obesity state. While leptin, the prototype adipokine, was increased in patients with breast malignancies and was positively associated with disease progression in vitro [4], serum adiponectin, a beneficial adipokine, was lower in breast cancer patients [5] and induced apoptosis and cell death in breast cancer cell lines [6].

With the increasing number of adipokines being discovered, novel molecules have been proposed to contribute to obesity-related carcinogenic mechanisms. One of them, omentin-1, is a novel adipokine with similar properties to adiponectin [7]. Omentin-1 treatment induced p53-dependent apoptosis of hepatocellular carcinoma cell lines in vitro [8]. In clinical studies, serum omentin-1 levels were increased in patients with prostate [9] and colorectal [10] cancer and decreased in patients with renal cell cancer [11] compared to controls. Most recently, *omentin* gene expression and circulating levels were lower in patients with breast cancer compared to healthy controls [12]. However, no previous studies have explored its levels in benign diseases of the breast or in response to neoadjuvant chemotherapy.

In addition, in the last decade, muscle tissue was also found to secrete hormone-like peptides, termed myokines [13]. Irisin, the best-known member of the myokine family, was initially proposed as a skeletal muscle-derived hormone, which is proteolytically cleaved from its precursor molecule termed fibronectin type III domain containing 5 (FNDC5) and is subsequently released into the circulation in response to exercise, improving glucose homeostasis by inducing browning of white fat thereby increasing its thermogenic activity [14]. Further studies have elucidated its multifaceted involvement in many pathophysiological diseases, though results are inconclusive. In particular, in vitro data have shown a possible anticarcinogenic profile of irisin in highly malignant breast cancer cell lines [15]. However, irisin protein expressions in breast cancer tissue specimens were higher compared to nonmalignant tissues [16]. In humans, irisin levels were lower in breast cancer patients compared to healthy controls, but in the same study, a positive association with tumor histology was shown [17]. Thus, the exact role of irisin in breast neoplasms is still poorly understood. Furthermore, it is currently unknown whether these molecules could be used as noninvasive diagnostic markers for the timely diagnosis of breast benign or to discriminate between benign growths and malignancies.

Therefore, the main aim of this study was to evaluate irisin and omentin-1 levels in patients with benign vs. malignant treatment-naïve and chemotherapy-treated breast neoplasms vs. apparently healthy controls and study associations with tumor aggressiveness features.

## 2. Subjects and Methods

### 2.1. Study Participants

Adult females attending the Breast Cancer Clinic, Department of Surgery, Theaghenio Cancer Hospital in Thessaloniki, Greece, due to recent diagnosis of highly suspicious breast growth and scheduled for a biopsy or surgical removal of the tumor were consecutively enrolled in the study, as previously described [18]. Subjects were subclassified as treatment-naïve cancer patients or individuals with benign breast diseases following histological examination of the tissue specimen. A subgroup of patients who had received neoadjuvant chemotherapy within the last year for reduction of tumor burden and scheduled for surgical removal of the residual tumor was additionally recruited to study the potential effects of neoadjuvant chemotherapy on the biomarkers of interest. Disease-free individuals, with no clinical and/or imaging evidence of breast neoplasms, followed up in the clinic as part of a breast cancer prevention program, were recruited as controls. Exclusion criteria were age less than 18 years, history of other malignancy of any origin, and presence of life-threatening, and muscle wasting diseases.

For all participants, demographic, anthropometric, and medical history data were individually recorded. Body composition parameters were assessed using bioelectrical impedance (BIA), as previously described [19].

After surgery, tumor pathology features including the presence of malignancy; tumor size (cm); the presence of local or disseminated disease; estrogen (ER), progesterone (PR), and human epidermal growth factor-2 (Her-2) receptors' status; Ki67 levels; and the Elston-Ellis modification of Scarff-Bloom-Richardson grading system [20] were recorded for cancer patients.

Early morning, fasting blood samples were collected the day before surgery for the benign and cancer group participants, and on a routine visit for the controls. The study protocol was approved by the Theagenio Cancer Hospital and the Aristotle University of Thessaloniki Ethics Committees in accordance with the Declaration of Helsinki. All participants provided a written informed consent.

### 2.2. Biochemical and Hormonal Measurements

Metabolic and biochemical parameters including serum glucose and lipid levels were measured using routine laboratory methods. Commercially available ELISA kits were used for the quantification of serum irisin (Phoenix Pharmaceuticals, Burlingame, CA, #EK-067-29, assay sensitivity 1.29 ng/ml, linear range 1.29–27.5 ng/ml) and omentin-1 (EMD Millipore Co., Burlington, MA, #EZH0MNTN1-29K, assay sensitivity 2 ng/ml). Intra-assay and interassay coefficients of variations were less than 10% for both assays.

### 2.3. Statistical Analysis

Statistical analysis of the data was performed with SPSS v20.0 for Windows (IBM Corp., Armonk, NY). Data for continuous variables are presented as mean ± standard deviation (SD), unless otherwise stated. The normality of distribution of the continuous variables was assessed with the Kolmogorov-Smirnov test. Not normally distributed variables were logarithmically transformed for comparisons, when appropriate. For between-group comparisons, the independent-*T* test or Mann–Whitney *U* test was performed in cases of two groups, and one-way analysis of variance (ANOVA) or Kruskal-Wallis test was used for between-group comparisons in cases of more than two groups, with post hoc Bonferroni correction, if needed. Pearson's or Spearman's correlation and partial coefficient was calculated for unadjusted and adjusted bivariate associations, respectively. Linear regression models were performed to identify independent predictors of continuous variables, e.g., tumor size. Univariate and multivariate logistic regression analyses were used to identify independent predictors of categorical outcomes, such as the presence of malignancy. To assess the diagnostic potential of the biomarkers of interest, we selected to run a number of comparisons between (1) cancer-free participants (control and benign groups) vs. treatment-naïve cancer patients to identify predictors of the presence of cancer in untreated populations, (2) cancer-free participants (control and benign groups) vs. all cancer patients (treatment-naïve and chemotherapy groups) to identify predictors of the presence of breast malignancy in the general population including participants under treatment, and (3) benign and treatment-naïve malignancy group to assess the potential of a hormone of interest to discriminate between a benign breast mass and breast malignancy. The level of statistical significance was set at 0.05 for all analyses.

## 3. Results

### 3.1. Descriptive Characteristics and Comparisons between Study Groups

Descriptive characteristics and case-control comparisons of the study variables are depicted in Table 1. Reflecting breast cancer epidemiology, healthy controls were younger compared to subjects of both cancer groups (*p* < 0.001 for both), but not the benign group. The percentage of total body fat (TBF%) obtained by BIA was significantly higher in the treatment-naïve cancer group compared to healthy individuals (*p*=0.03). Regarding biochemical markers, we found significantly lower glucose levels in the disease-free control group compared to both treatment-naïve (*p*=0.02) and chemotherapy-treated (*p*=0.001) cancer groups and lower glucose levels in the benign group compared to the chemotherapy-treated group only (*p*=0.03). Total cholesterol in both cancer groups was higher than that in the control group (*p*=0.04 for both), but not the benign group (Table 1).

**Figure 1:**
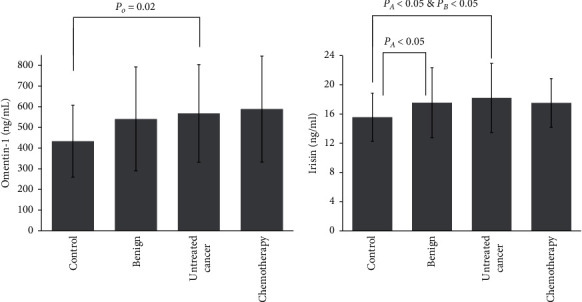
Omentin-1 (a) and irisin (b) serum levels across study groups. Post hoc unadjusted and adjusted comparisons were employed. Analysis of variance (ANOVA) or Kruskal-Wallis tests were used in unadjusted comparisons. Analysis of covariance (ANCOVA) was used in adjusted comparisons. Logarithmically transformed variables were used for both molecules. Adjusted models included: Model 1: adjusted for age. Model 2: adjusted for age and Ln-Body Mass Index (BMI). Model 3: adjusted for age, Ln-BMI and glucose levels.  Model 4: adjusted for age, Ln-BMI, glucose levels, and total cholesterol levels. Model 5: adjusted for age, Ln-BMI, glucose levels, total cholesterol levels, and Ln-omentin-1. *P*_*O*_: control significantly lower (*p* < 0.05) compared to treatment-naïve cancer group only (in the unadjusted model). *P*_*A*_: control significantly lower (*p* < 0.05) compared to both benign and treatment-naïve cancer group (Model 1- Model 4). *P*_*B*_: control significantly lower (*p* < 0.05) compared to treatment-naïve cancer group only (in unadjusted model and Model 5).

In raw comparisons (overall p for trend: *p*=0.01), treatment-naïve cancer group patients had significantly higher logarithmically (Ln) transformed omentin-1 levels compared to healthy controls (*p*=0.02) (Figure 1(a)). Post hoc comparison between healthy control and chemotherapy-treated group subjects was borderline significant (*p*=0.06), but significance was lost after adjustment for age (*p*=0.13), and this result remained unaffected after further adjustment for Body Mass Index (BMI), serum glucose, and total cholesterol (*p* for trend >0.05 for all) (Figure 1(a)).

Furthermore, regarding irisin (overall *p* for trend *p* < 0.01) treatment-naïve cancer group patients exhibited higher Ln-transformed irisin levels compared to healthy controls (*p* < 0.01) (Figure 1(b)). Unadjusted post hoc comparison between healthy controls and the benign group was borderline significant (*p*=0.07). However, adjustment for age (Model 1, overall p for trend: *p* < 0.01) resulted in a significant pairwise comparison between healthy controls and the benign group participants (*p*=0.04). The significant comparison between the control group and treatment-naïve cancer group remained unaffected (*p*=0.001). Further adjustment for BMI (Model 2, overall *p* for trend: *p* < 0.01) did not impact significance. When glucose levels were added to the model (Model 3-overall p for trend: *p*=0.001), irisin concentrations in the control group were significantly lower compared to both benign (*p*=0.02) and treatment-naïve groups (*p*=0.001). The same results were evident when BMI was replaced with TBF% in Model 3, or when total cholesterol was added to the model (Model 4, overall *p* for trend: *p*=0.01), i.e., *p*=0.04 for benign and *p* < 0.01 for treatment-naïve cancer group vs. controls, respectively. Interestingly, when Ln-transformed omentin-1 levels were added to Model 4 (Model 5, overall *p* for trend: *p*=0.02), with either BMI or TBF%, in post hoc pairwise comparisons, irisin levels in the control group remained significantly lower compared to the treatment-naïve cancer group only (*p*=0.02) (Figure 1(b)).

### 3.2. Associations of the Molecules of Interest with Other Study Variables

Both hormone levels were similar in patients with vs. without hypertension, with vs. without hyperlipidemia, with vs. without diabetes mellitus, with vs. without chronic kidney disease, with vs. without the chronic obstructive pulmonary disease, and with vs. without a history of pregnancies, breastfeeding, and/or history of familial breast cancer or malignancy of any origin (*p* > 0.05 for all).

In the whole cohort, Pearson's bivariate coefficient analysis revealed a positive association between irisin and omentin-1 (*r* = 0.28, *p* < 0.001). Positive associations between omentin-1 and age (*r* = 0.26, *p* < 0.001), WHR (*r* = 0.19, *p*=0.01), and glucose levels (*r* = 0.15, *p*=0.04) were also found (Table 2).

Partial correlation coefficients were performed to identify associates of the study variables after adjusting for potential confounders (Table 2). Adjustment for age did not affect the positive association between irisin and omentin-1 (*r* = 0.29, *p* < 0.001) but resulted in a significant negative association between omentin-1 and BMI (*r* = −0.19, *p*=0.01). Significance in the correlation between omentin-1 and WHR was initially lost but reestablished after further controlling for BMI and blood glucose (Table 2). The strong positive association between irisin and omentin-1 remained unaffected even after further adjustments for BMI and/or serum glucose levels (*p* < 0.001 for all) (Table 2).

### 3.3. Logistic Regression Analyses to Identify Predictors of the Presence of Cancer

Univariate and multivariate models were used to identify independent predictors of the presence of malignancy in a number of comparisons. In the pooled group analysis comparing cancer-free participants to patients with either recently identified or known breast malignancy under treatment, the only independent predictors were age (odds ratio (OR) = 1.05, 95% confidence interval (95% CI) = (1.02, 1.08), *p* < 0.01) and irisin levels (OR = 5.30, 95% CI = (1.24, 22.38), *p*=0.03) (Table 3). Similar results were observed when cancer-free individuals were compared to the recently identified treatment-naïve cancer group, i.e., for age (OR = 1.04, 95% CI = (1.01, 1.08), *p*=0.02) and for irisin (OR = 5.05, 95% CI= (1.14, 22.40), *p*=0.03) (Table 3). However, there were no independent predictors that could discriminate between benign mass and malignancies (Table 3).

### 3.4. Associations with Tumor Histological Features in the Treatment-Naïve Cancer Group

**Figure 2:**
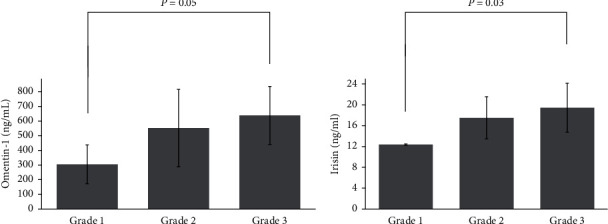
Omentin-1 (a) and irisin (b) serum levels according to the Elston-Ellis modification of the Scarff-Bloom-Richardson grading system. Data are presented as mean ± SD. Analysis of variance (ANOVA) and post hoc comparisons were employed. Logarithmically transformed variables were used for both molecules.

Regarding associations with tumor aggressiveness characteristics, in the treatment-naïve cancer group, omentin-1 was associated with neither tumor size nor Ki67% levels, whereas irisin was positively correlated with the latter (*r* = 0.28, *p*=0.03). Both hormones showed an increasing pattern with the Elston-Ellis grading system, i.e., for omentin-1, Grade 1: 304.61 ± 132.26 ng/ml vs. Grade 2: 530.65 ± 243.29 ng/ml vs. Grade 3: 661.61 ± 204.25 ng/ml, *p*=0.01 (with borderline significantly lower levels in Grade 1 compared to Grade 3, *p*=0.05) (Figure 2(a)) and for irisin, Grade 1: 12.37 ± 0.14 ng/ml vs. Grade 2: 17.40 ± 4.24 ng/ml vs. Grade 3: 20.04 ± 5.08 ng/ml, *p*=0.01 (with post hoc Bonferroni correction showing significantly lower levels in Grade 1 compared to Grade 3, *p*=0.03) (Figure 2(b)). No differences were observed regarding progesterone and Her-2 receptors status for both molecules. However, patients with ER+ breast cancer exhibited lower irisin levels compared to ER-breast cancer patients (ER+: 17.84 ± 4.84 ng/ml vs. 19.99 ± 3.69 ng/ml, *p*=0.04).

## 4. Discussion

Obesity is now considered a major modifiable risk factor for cancer development [21]. Therefore, understanding the mechanisms that link obesity with carcinogenesis is important for the identification of novel diagnostic markers and/or therapeutic targets. In this study, we explored serum levels of muscle- and adipose-tissue-derived molecules, namely, irisin and omentin-1, in patients with benign and malignant breast neoplasms of different stages compared to healthy individuals. We showed that serum irisin concentrations are increased in patients with both benign and malignant proliferative diseases of the breast independently of potential confounders and are also associated with tumor aggressiveness. In the fully adjusted model, adjusting for omentin-1 showed that irisin was an independent predictor of the presence of malignancy when compared to cancer-free individuals.

Irisin is a recently discovered adipomyokine that was initially suggested as a browning factor of white adipose tissue, orchestrating the beneficial effects of exercise in metabolism, and, thus, raising high expectations as a potential therapeutic agent against metabolic diseases [14]. In follow-up studies, our group was one of the first to elucidate irisin regulation in humans [22, 23]. However, increasing controversy regarding the accuracy and specificity of irisin assays has later emerged, questioning previous findings [24, 25].

Regarding cancer studies, irisin's role in carcinogenesis was recently debated [26]. Preliminary in vitro data showed no effect of irisin treatment in the colon, thyroid, esophageal, and endometrial cancer cell lines [27], while later data supported potential anticarcinogenic properties of irisin in malignant breast [15], lung [28], osteosarcoma [29], and pancreatic [30] cancer cell lines. In contrast, in a recent study, it was found that irisin might increase cell proliferation and migration potential of human hepatocellular carcinoma (HCC) cells [31]. Interestingly, in an animal study, FNDC5 gene expression in white adipose tissue and serum irisin levels were increased in mice with experimentally induced gastric cancer, compared to noncancer or control groups [32]. In human studies, FNDC5 hepatic gene expression was higher in HCC patients undergoing liver transplantation compared to deceased donors [33]. Protein expression of FNDC5/irisin was higher in breast and ovarian cancer tissue specimens [16], as well as in oncocytic papillary thyroid carcinoma [34] and in a series of gastrointestinal cancers compared to nonneoplastic tissues [35]. In addition, elevated irisin expression was observed in cancer cells and tumor stromal fibroblasts of non-small-cell lung carcinoma [36]. Furthermore, in clinical studies recruiting colorectal [37], prostate [38], and bladder [39] cancer patients, serum irisin levels were lower compared to healthy controls. Conversely, increased irisin concentrations were found in renal cancer patients compared to disease-free individuals [40].

Regarding breast cancer, it was suggested that cancer patients exhibit low irisin concentrations [17] and irisin is also protective against metastatic disease [41]. However, in the former study, the ELISA kit used reported irisin levels 100-fold higher than our observations, and based on previous validations, it is less specific. In contrast, the ELISA kit we have used herein has been validated with Tandem Mass Spectrometry [42]. In that study, irisin concentrations were reported in the range of 3–5 ng/ml [42] which is lower than the values reported herein. However, our findings are of a similar magnitude with other published studies using the same ELISA kit [43–46] and indeed slightly higher than the Tandem Mass Spectrometry but significantly lower compared to assays used in different studies. In fact, our assay's linear range is 1.29–27.5 ng/ml, and the assay can detect irisin levels between 10 and 50 ng/ml. Values reported in the present study fall well within this range. In addition, Jedrychowski et al. assessed irisin levels in young, healthy, and normal-weight individuals [42] which is different from our study sample recruiting older individuals with higher BMI and other metabolic comorbidities, which are generally positively associated with higher irisin concentrations [47, 48]. In any case, as irisin was measured with the same kit in all groups and as the conclusions of our study were based on the observed differences of irisin concentrations between the groups, the actual irisin values do not influence the interpretation of the results.

Moreover, in accordance with our observations, a positive association with tumor histology was observed [17]; the authors did not recruit patients with benign diseases of the breast and suggested that these controversial results might be partly explained due to complex and less understood pathophysiological changes occurring in malignancy. In our study recruiting patients with benign diseases of the breast as well as cancer patients that had received neoadjuvant chemotherapy, we found significantly higher serum irisin levels in both benign and newly diagnosed breast cancer subjects compared to healthy individuals after adjusting for potential covariates and a positive association with tumor behavior. We also showed that irisin concentrations could independently predict the presence of malignancy in the general population. Our results are consistent with immunohistochemistry studies, and discrepancies with other serum studies may be explained by the use of less-validated methods to assess irisin concentrations as well as differences in study design and study sample selection. Our novel findings indicate a possible involvement of FNDC5/irisin in breast tumorigenesis, even from very early stages of development of benign growths, as well as in processes of cancer progression, thereby pointing toward its use as a potential diagnostic and prognostic marker for breast neoplasms; this, however, needs to be confirmed in future prospective studies. When we adjusted our analyses for omentin-1 levels, a surrogate marker of visceral obesity, the comparison between healthy controls and patients with benign tumors failed to reach significance while comparison with cancer patients was persistent, suggesting that the association between irisin concentration and presence of breast malignancy is not affected by visceral adiposity. However, this needs to be confirmed in larger cohorts and/or future longitudinal studies.

Omentin-1, initially termed as intelectin-1, was suggested as visceral fat-derived adipokine with anti-inflammatory and insulin-sensitizing properties [49] which is reduced in obesity, in a similar manner with adiponectin, and drives the higher burden of excessive body fat in patients with increased cardiovascular risk or type 2 diabetes [7]. In a previous study recruiting a rather limited group of individuals at higher cardiovascular risk, we found that omentin-1 was negatively associated with irisin and also closely associated with lipoprotein subparticle profile, possibly indicating a role as a marker of increased cardiovascular risk [50]. These results are not directly comparable to those of the present study, in which we found a positive association between these two molecules, due to differences in the study sample characteristics and study aims. Therefore, this association requires further research in future studies with larger populations. Regarding cancer, although in vitro data have shown possible anticarcinogenic effects of this molecule, findings from clinical studies were contradictory, with most studies showing increased omentin-1 serum concentrations in cancer patients compared to disease-free controls (as reviewed in [51]). However, in a very recent study, omentin gene expression and circulating levels were lower in patients with breast cancer compared to healthy controls [12]. However, to the best of our knowledge, no previous study has assessed omentin-1 levels across the spectrum of proliferative diseases of the breast. Therefore, we have conducted the present study in which we found that omentin-1 levels are increased in treatment-naïve breast cancer patients compared to healthy individuals in unadjusted comparisons. These results are in accordance with a meta-analysis suggesting that malignancies are generally associated with increased omentin-1 levels [52]. However, in our study, the significance of this comparison did not persist after adjustments for covariates, indicating that anthropometric and metabolic parameters, rather omentin-1, are the main predictors of the presence of malignancy. In addition, we reported that serum omentin-1 levels are increased in patients with more aggressive disease burden based on the Elston-Ellis grading system, which may be explained by activation of Akt signaling pathways and/or inflammatory processes [10].

The strengths of this study include its novelty and the simultaneous assessment of two hormones with promising metabolic properties in a group of subjects with breast neoplasms with a wide age range. To the best of our knowledge, this is the first study to explore serum irisin and omentin-1 levels across the spectrum of breast neoplasms, recruiting patients with benign breast lesions as well as newly diagnosed, treatment-naïve cancer patients and individuals who received neoadjuvant chemotherapy and were scheduled for surgical removal of the residual tumor, comparing their levels to healthy controls. We showed that irisin is elevated in either benign or malignant breast diseases, independently of possible covariates. In addition, we have assessed irisin levels with a Tandem Mass Spectrometry-validated kit and examined associations with several aspects of tumor histology. However, due to the case-control design of our study, the causality of the reported associations cannot be supported. However, our results provide an impetus for the design and interpretation of larger studies exploring the involvement of these adipomyokines in neoplasms of the breast. Our rather small study sample may also be considered as a limitation and, as patients were recruited from a single center, a selection bias may be committed. Therefore, larger multicenter studies are needed to further elucidate the role of irisin and/or omentin-1 in breast tumorigenesis.

## 5. Conclusions

In conclusion, we reported for the first time that serum irisin is increased in both benign and malignant neoplasms compared to healthy controls and, after controlling for potential confounding factors including omentin-1, irisin was associated with breast cancer presence. Irisin was also associated with tumor aggressiveness and may, therefore, hold potential as a future diagnostic and/or prognostic marker. Our results warrant further investigation in future mechanistic and longitudinal studies and/or clinical trials.

## Figures and Tables

**Table 1:** Descriptive characteristics of the study variables and unadjusted case-control comparisons.

Variable	Control group	Benign group	Treatment-naïve cancer group	Chemotherapy cancer group	*p* value for trend
*N*	56	61	72	24	
Age (years)	48.05 ± 10.79^	52.25 ± 12.28	57.61 ± 12.29	61.21 ± 10.64	**<0.001**
Weight (kg)	71.08 ± 12.59	69.85 ± 13.12	74.39 ± 14.57	74.26 ± 13.94	0.21
Height (cm)	162.20 ± 5.84	159.84 ± 6.97	161.22 ± 7.13	156.88 ± 6.23	**0.01**
Body Mass Index (kg/m^2^)	27.38 ± 5.18	27.45 ± 5.52	28.59 ± 5.15	30.14 ± 5.16	0.07
Total body fat (%)	34.90 ± 7.01^∗^	36.77 ± 8.28	39.30 ± 9.98	39.00 ± 8.40	**0.03**
Waist-hip-ratio	0.86 ± 0.71	0.86 ± 0.77	0.87 ± 0.67	0.89 ± 0.57	0.27
Glucose (mg/dL)	94.48 ± 10.35 ^	100.37 ± 17.32^#^	105.70 ± 23.45	115.24 ± 33.80	**0.01**
Triglycerides (mg/dL)	104.27 ± 49.49	98.89 ± 47.93	106.21 ± 55.80	118.76 ± 39.60	0.26
Total cholesterol (mg/dL)	207.89 ± 39.79 ^	220.77 ± 37.18	208.04 ± 35.92	236.14 ± 50.90	**0.02**

Data are presented as mean ± standard deviation (SD). ANOVA or Kruskal-Wallis tests were used in unadjusted comparisons. Post hoc comparisons were determined with Bonferroni correction. For comparisons, logarithmically transformed variables were used for Body Mass Index and triglycerides. Significance is highlighted in bold. ^*∗*^Control significantly lower (*p* < 0.05) compared to the treatment-naïve group only (in the unadjusted model): control significantly lower (*p* < 0.05) compared to the benign and treatment-naïve group. ^Control significantly lower (*p* < 0.05) compared to both treatment-naïve and chemotherapy-treated cancer group. #Benign significantly lower (*p* < 0.05) compared to chemotherapy-treated cancer group.

**Table 2:** Bivariate correlation matrices. Unadjusted Pearson's or Spearman's coefficients and partial correlation coefficient after sequential adjustment for age, Body Mass Index (BMI), and glucose levels in the overall study group.

	OMENTIN-1 (ng/mL)				IRISIN (ng/mL)			
	Unadjusted	Adjusted for age	Adjusted for age & BMI	Adjusted for age, BMI & glucose levels	Unadjusted	Adjusted for age	Adjusted for age & BMI	Adjusted for age, BMI & glucose levels
	*R*		*R*	*R*	*R*	*R*		*R*
Age (years)	0.26^*∗∗∗*^	**—**	**—**	**—**	0.01	**—**	**—**	**—**
Weight (kg)	−0.07	−0.14	0.06	0.06	0.01	−0.04	−0.04	−0.04
Height (cm)	−0.01	0.07	−0.05	0.05	−0.03	−0.06	−0.06	−0.06
Body Mass Index (kg/m^2^)^#^	−0.07	−**0.19^∗^**	**—**	**—**	0.01	−0.02	**—**	**—**
Total body fat (%)	−0.01	−0.14	−0.01	0.01	0.02	−0.03	−0.02	−0.02
Waist-hip-ratio	0.19^*∗∗*^	0.13	**0.18** ^*∗*^	**0.18** ^*∗*^	0.11	0.06	0.06	0.07
Glucose (mg/dL)	0.15^*∗*^	0.02	0.08	**—**	−0.05	−0.12	−0.11	**—**
Triglycerides (mg/dL)^#^	−0.01	−0.13	−0.08	−0.08	−0.09	−0.08	−0.08	−0.08
Total cholesterol (mg/dL)	−0.04	−0.04	−0.04	−0.03	0.09	0.09	0.09	0.06
Omentin 1 (ng/mL)^#^	**—**	**—**	**—**	**—**	**0.28** ^*∗∗∗*^	**0.29** ^*∗∗∗*^	**0.29** ^*∗∗∗*^	**0.30** ^*∗∗∗*^
Irisin (ng/mL)^#^	0.28^*∗∗∗*^	0.29^*∗∗∗*^	0.29^*∗∗∗*^	0.30^*∗∗∗*^	**—**	**—**	**—**	**—**

Significant correlations are highlighted in bold. ^*∗*^*p* < 0.05, ^*∗∗*^*p* < 0.01, and ^*∗∗∗*^*p* < 0.001. ^#^Logarithmically transformed variables were used for analyses.

**Table 3:** Independent predictors for the presence of malignancy.

	Univariate		Multivariate	
	OR (95% CI)	*p*	OR (95% CI)	*p*
(a) Comparison between cancer-free (controls and benign group individuals) vs. cancer (treatment-naïve and chemotherapy-treated) combined groups.				
Age (years)	1.06 (1.03, 1.09)	**<0.001**	1.05 (1.02, 1.08)	**<0.01**
BMI (kg/m^2^)^*∗*^	6.01 (1.29, 28.07)	**0.02**		
TBF (%)^*∗*^	4.21 (1.28, 13.80)	**0.02**		
Glucose (mg/dL)	1.03 (1.01, 1.04)	**0.001**		
Total cholesterol (mg/dL)	1.00 (0.99, 1.01)	0.90		
Omentin-1 (ng/mL)^*∗*^	2.23 (1.18, 4.21)	**0.01**		
Irisin (ng/mL)^*∗*^	4.81 (1.42, 16.23)	**0.01**	5.30 (1.24, 22.69)	**0.03**

(b) Comparison between cancer-free (controls and benign group individuals) vs. treatment-naïve cancer group.				
Age (years)	1.06 (1.03, 1.08)	**<0.001**	1.04 (1.01, 1.08)	**0.02**
BMI (kg/m^2^)^*∗*^	3.81 (0.74, 19.52)	0.11		
TBF (%)^*∗*^	4.46 (1.20, 16.61)	**0.03**		
Glucose (mg/dL)	1.03 (1.01, 1.04)	**0.01**		
Total cholesterol (mg/dL)	0.99(0.99, 1.004)	0.30		
Omentin-1 (ng/mL)^*∗*^	2.07 (1.04, 4.1 1)	**0.04**		
Irisin (ng/mL)^*∗*^	4.98 (1.38, 18.03)	**0.01**	5.05 (1.14, 22.40)	**0.03**

(c) Comparison between benign group individuals vs. treatment-naïve cancer group.				
Age (years)	1.04 (1.01, 1.07)	**0.02**	1.03 (0.99, 1.07)	0.10
BMI (kg/m^2^)^*∗*^	3.83 (0.56, 26.44)	0.17	1.84 (0.05, 66.32)	0.74
TBF (%)^*∗*^	2.69 (0.66, 10.93)	0.17	1.07 (0.09, 13.24)	0.96
Glucose (mg/dL)	1.01 (0.995, 1.03)	0.16	1.00 (0.98, 1.03)	0.76
Total cholesterol (mg/dL)	0.99(0.98, 1.001)	0.07	0.99 (0.98, 1.004)	0.19
Omentin-1 (ng/mL)^*∗*^	1.30 (0.62, 2.74)	0.49	0.79 (0.33, 1.87)	0.59
Irisin (ng/mL)^*∗*^	1.95 (0.46, 8.23)	0.36	1.65 (0.25, 10.98)	0.60

Multivariate model includes age, glucose, BMI, %TBF, total cholesterol levels, serum omentin-1, and serum irisin levels. Significance is highlighted in bold. OR: Odds Ratio, BMI: Body Mass Index, TBF: total body fat. ^*∗*^Logarithmically transformed values were used for comparisons.

## Data Availability

Anonymized datasets used to support the findings of the present manuscript are available from the corresponding author, upon reasonable request.
